# A reference genome for Bluegill (Centrarchidae: *Lepomis macrochirus*)

**DOI:** 10.1093/g3journal/jkad019

**Published:** 2023-01-23

**Authors:** William B Ludt, Eamon C Corbett, Jerry Kattawar, Prosanta Chakrabarty, Brant C Faircloth

**Affiliations:** Department of Ichthyology, Natural History Museum of Los Angeles County, Los Angeles, CA 90007, USA; Museum of Natural Science and Department of Biological Sciences, Louisiana State University, Baton Rouge, LA 70803, USA; Museum of Natural Science and Department of Biological Sciences, Louisiana State University, Baton Rouge, LA 70803, USA; Museum of Natural Science and Department of Biological Sciences, Louisiana State University, Baton Rouge, LA 70803, USA; Museum of Natural Science and Department of Biological Sciences, Louisiana State University, Baton Rouge, LA 70803, USA

**Keywords:** Bluegill, sunfish, *Lepomis macrochirus*, Centrarchidae, Lepominae, bass, bream, perch, nanopore sequencing, HiC

## Abstract

North American sunfishes (Family Centrarchidae) are among the most popular sportfish throughout the United States and Canada. Despite the popularity of sunfishes, their ecological importance, and their extensive stocking and aquacultural history, few molecular studies have examined the evolutionary relationships and species boundaries among members of this group, many of which are known to hybridize. Here, we describe a chromosome-scale genome assembly representing Bluegill (*Lepomis macrochirus*), one of the most widespread centrarchid species. By combining long-read, Oxford Nanopore sequencing data with short-insert, whole-genome and HiC sequence reads, we produced an assembly (Lm_LA_1.1) having a total length of 889 Mb including 1,841 scaffolds and having a scaffold N50 of 36 Mb, L50 of 12, N90 of 29 Mb, and L90 of 22. We detected 99% (eukaryota_odb10) and 98% (actinopterygii_odb10) universal single-copy orthologs (BUSCOs), and ab initio gene prediction performed using this new assembly identified a set of 17,233 genes that were supported by external (OrthoDB v10) data. This new assembly provides an important addition to the growing set of assemblies already available for spiny-rayed fishes (Acanthomorpha), and it will serve as a resource for future studies that focus on the complex evolutionary history of centrarchids.

## Introduction

**Fig. 1.**
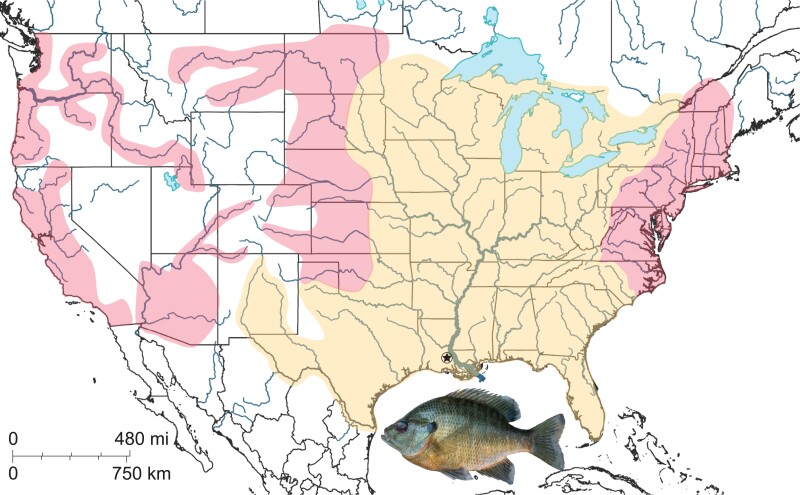
The native (in orange) and introduced (red) range of the Bluegill (*L. macrochirus*) in the United States. The vouchered individual used in this study is pictured (LSUMZ 21031), and its collection location is marked with a star on the map.

North American sunfishes (Family Centrarchidae) constitute one of the most popular recreational fisheries in North America (Page and Burr 2011). Sunfishes are also ecologically important freshwater predators (Aday *et al.* 2009). The family contains 38 species distributed among 8 genera (Fricke *et al.* 2022) and includes well-known fishes such as largemouth and other black bass (*Micropterus* spp.), crappies (*Pomoxis* spp.), and sunfish (*Lepomis* spp.). Aside from the California endemic Sacramento Perch, *Archoplites interruptus* (Girard 1854), sunfishes are native east of the Rocky Mountains, and their range extends north into Canada and south into northern Mexico (Page and Burr 2011). The most widespread sunfish species is Bluegill, *Lepomis macrochirus* (Rafinesque 1819), which are native to the St. Lawrence/Great Lakes region and basins of the Mississippi River down to the Gulf of Mexico (Fig. 1). Bluegill are also native to drainages along the southern Atlantic Coast of North America in the east and the Rio Grande drainage in Texas and Mexico to the west (Page and Burr 2011). Bluegill have been introduced outside of their native range to the western United States and also to localities including Africa (Ndaleni *et al.* 2018), Oceania (Yamamoto 1992), and Asia (Kawamura *et al.* 2006), where they have created management problems due to competition with native species (Maezono and Miyashita 2003).

Despite the widespread popularity of sunfishes, their ecological importance, and their aquacultural history (Regier 1962), few molecular studies examining the evolutionary relationships and species limits within centrarchids have occurred during the past 20 years (Near *et al.* 2004, 2005; Near and Kim 2021). As such, genomic resources for this group, including genome assemblies, are lacking for all species except for 3 species of black bass (Supplementary Table 1; Sun *et al.* 2021).

Although genome data for sunfishes are few, genome-enabled studies would significantly advance our understanding of the group. For example, some sunfishes of the genus *Lepomis* display considerable color and meristic variation across their ranges, and these variable traits have historically caused taxonomic confusion (Near and Koppelman 2009). Whole-genome resequencing data aligned to a high-quality reference assembly for 1 or more species of sunfish would help clarify our understanding of species limits in the group while also enabling studies of the genomic basis for these incredibly variable traits. Hybridization is also common among centrarchids, with 31 species pairs known to hybridize in the wild (Bolnick 2009) and reports of hybrids between species of different genera (Burr 1974). Yet, the extent of introgression between species, the effects of introgression on the delineation of species boundaries, and the role of introgression on species diversity in this group are largely unknown—a high-quality reference assembly for sunfishes would enable these studies. Finally, under certain circumstances, Bluegill diverge into pelagic and benthic ecomorphs (Uchii *et al.* 2007), similar to Threespine Sticklebacks (*Gasterosteus aculeatus*; Rundle *et al.* 2000), and a high-quality reference assembly for this species would provide an important comparative resource for studying the evolution of these forms.

Here, we expand the genomic resources available for Centrarchidae by describing a chromosome-scale assembly (Lm_LA_1.1) we produced for a vouchered (Buckner *et al.* 2021), male Bluegill collected from Louisiana.

## Methods

We collected muscle, gill, fin, and liver tissues from a male Bluegill captured at the Sherburne Wildlife Management Area (30.515441, −91.7164) during 2018 under Louisiana Department of Fish and Wildlife Collecting Permit SCP167 and LSU IACUC 18-065. Tissues were flash-frozen immediately in the field. After tissue collection, we prepared a specimen for the LSU Museum of Natural Science (LSUMNS) Collection of Fishes (LSUMZ 21031), and we stored tissue samples from this specimen in the LSUMNS Collection of Genetic Resources (LSUMZ 10149). We subsampled ∼25 mg of gill tissue to prepare a short-insert library for this individual, and we subsampled and shipped ∼25–30 mg of liver tissue to Dovetail Genomics to prepare and sequence long-read and HiC libraries.

We extracted DNA from the 25-mg subsample of gill tissue using a Qiagen DNeasy extraction kit and quantified extracted DNA using a fluorometer (Life Technologies, Inc.). After quantification, we sheared 650 ng DNA to a modal size of 500–600 bp using a sonicator (Qsonica, Newtown, CT, USA; 12 cycles of 20 s on and 20 s off), and we input 250 ng of sheared DNA to a commercial library preparation kit (Kapa HyperPrep; F. Hoffmann-La Roche AG) following the PCR-free protocol to incorporate unique dual index adapters (Integrated DNA Technologies, Inc.). Following library preparation, we performed a 1.8× (v/v) SPRI bead cleanup (Rohland and Reich 2012) followed by a column-based cleanup (Qiagen GeneRead Size Selection Kit) and quantified the cleaned product using a fluorometer. Then, we determined the insert size distribution of the library using a Bioanalyzer (Agilent, Inc.) and quantified the library using a commercial qPCR kit (Kapa Library Quantification Kit; F. Hoffmann-La Roche AG). We sequenced the library as part of a paired-end (PE), 150 bp lane of Illumina NovaSeq (Novogene, Inc.), targeting ∼50× coverage after assuming a genome size of ∼1 Gb (Ragland and Gold 1989). After sequencing, we used jellyfish (v2.3.0; Marcais and Kingsford 2011) to count kmers (kmer size = 21), and we input the kmer histogram to GenomeScope (Vurture *et al.* 2017) to estimate genome size.

Dovetail staff extracted DNA from a subsample of the liver tissue shipped to their facility following the Qiagen Genomic DNA extraction protocol for tissues (QIAGEN 2015) and using a Qiagen Tip-100 Midi Column, and they prepared Oxford Nanopore 1D libraries (Rapid Sequencing Kit SQK-RAD004) from extracted DNA with slight modifications to the protocol. Modifications included using variable amounts of input DNA (3–4 µg), using smaller amounts of fragmentation mix (1–2.5 µl), and extending the ligation time to 20 min for most reactions (Supplementary Table 1). After preparation, long-read libraries were sequenced on an Oxford Nanopore MinION using an R9.4 flowcell, and basecalling was performed using MinKnow 1.15.1 (Oxford Nanopore Technologies PLC). Data were generated from all libraries to achieve an approximate depth of 33× assuming a genome size of 1 Gbp. Dovetail staff also prepared 3 HiC libraries following a protocol similar to that described in Lieberman-Aiden *et al.* (2009) and summarized in Salter *et al.* (2019), and they generated data from each HiC library using PE 150 BP sequencing on an Illumina HiSeq X targeting 150–250 million read pairs per library.

We assembled the long-read FASTQ data received from Dovetail using wtdbg2 (v2.5; Ruan and Li 2020) and flye (v2.9-b1774; Kolmogorov *et al.* 2019) on a 1.5 TB RAM compute node and computed contiguity and completeness metrics of the assemblies using assembly-stats (Wellcome Sanger Institute 2022a) and BUSCO (eukaryota_odb10; Manni *et al.* 2021). The BUSCO results suggested that flye produced a more complete assembly, so we ran 2 additional rounds of long-read polishing in flye (for a total of 3), and we used the resulting flye assembly in all subsequent steps. We performed 1 round of short-read polishing by trimming the adapters and low-quality bases from the short insert, Illumina data using trimmomatic, aligning the trimmed data to the flye contigs using BWA (v0.7.17; Li 2013) and SAMtools (v1.10; Li *et al.* 2009), and using Pilon (v1.23; Walker *et al.* 2014) to fix “–all” of the issues identified (where possible).

After short-read polishing, we trimmed the HiC libraries for adapters and low-quality bases using trimmomatic, we combined all trimmed read files, and we used the juicer workflow (v1.6; Durand, Shamim, *et al*. 2016) to align the trimmed HiC data to the polished assembly, remove duplicates, and compute HiC library metrics. Then, we generated temporary scaffolds using 3D-DNA (v180922; Dudchenko *et al.* 2017) with error correction turned off, manually corrected the temporary scaffolds using JuiceBox (v1.11.08; Durand, Robinson, *et al*. 2016) where the HiC contact map suggested a misjoin, and rescaffolded the assembly using the 3D-DNA post-review assembly workflow. To improve the orientation of contigs within scaffolds, we ran the resulting assembly through HiCHiker (v1.0.0; Nakabayashi and Morishita 2020), and we used faFilter (Kent *et al.* 2002) to remove contigs/scaffolds from the assembly that were shorter than 1,000 bp. We also used BlobTools (v2.6.3; Laetsch and Blaxter 2017) to compute (long read) coverage of the assembly, perform taxonomic partitioning of the scaffolds/contigs, and remove scaffolds having <5× coverage. We mapped 1 library of HiC read pairs (DTG-HiC-732) to the remaining scaffolds and contigs using the Arima Genomics Mapping Pipeline (commit b001ebc; Arima Genomics 2019), BWA (v0.7.17), and SAMtools (v1.10), and we used PretextMap (v0.1.9) and PretextView (v0.2.5; Wellcome Sanger Institute 2022b) to produce a visual representation of the contact map.

After removing low-coverage contigs, we used the Dfam TE Tools container (v1.3.1; Dfam-Consortium 2022) to run RepeatModeller (v2.0.2; Flynn *et al.* 2020) identification of transposable elements (including the “-LTRStruct” option), and we input the repeat models to RepeatMasker (v4.1.2-p1; Smith *et al.* 2022). We used the general feature format file output by RepeatMasker with BEDTools (v2.17.0; Quinlan and Hall 2010) to soft mask the assembly. After soft-masking, we renamed the scaffolds and contigs; sorted the contigs and scaffolds by name/size using SeqKit (v2.2; Shen *et al.*^2016^); removed 1 scaffold that represented a long, improperly linearized version of the *L. macrochirus* mitochondrial genome that we identified using a minimap2 (v2.17-r941; Li 2018) alignment to the *L. macrochirus* mitochondrial RefSeq (NC_015984.2); and computed a final set of contiguity and completeness metrics using assembly-stats and BUSCO (with both eukaryota_odb10 and actinopterygii_odb10 databases). We also estimated assembly completeness and consensus quality value (QV) by counting kmers in short insert, Illumina data using meryl (v1.3) with a *k*-value of 20 and inputting the meryl database, along with the final version of the assembly, to Merqury (v1.3; Rhie *et al.* 2020). To recover a properly linearized assembly of the mitochondrial genome, we input the long read and the short insert, Illumina data to mitoVGP (v2.2; Formenti *et al.*^2021^).

Finally, we performed a single round of ab initio gene prediction using a containerized build (Faircloth 2022) of Braker2-GenemarkEP+-Augustus (v2.1.6; Lomsadze *et al.* 2005; Stanke *et al.* 2006, 2008; Gotoh 2008; Iwata and Gotoh 2012; Buchfink *et al*. 2015; Hoff *et al.* 2016, 2019; Brůna *et al.* 2020, 2021) and a file of vertebrate protein sequences from OrthoDB v10 (Kriventseva *et al.* 2019). We functionally annotated the predicted protein sequences output by Braker2 using InterProScan (v5.57-90.0; Jones *et al.* 2014), and we used an accessory script from the braker2 repository (selectSupportedSubsets.py), along with custom Python code, to produce a filtered version of the predicted transcript sequences that were “fully supported” by external evidence. The braker2 accessory script describes “fully supported” gene sequences as those transcripts: (1) that are complete, (2) where all introns in a transcript are supported by external (OrthoDB protein) evidence, and (3) that have start and stop codons supported by external (OrthoDB protein) evidence, when transcripts are composed of a single exon.

## Results and discussion

Illumina sequencing of the short-insert library produced 195,820,475 read pairs with an average insert size of 467 bp. GenomeScope results using a kmer size of 21 estimated that the length of the haploid Bluegill genome was 0.751–0.752 Gb, suggesting that the short-insert reads approximated 78× coverage. Eight flowcells of nanopore sequencing produced a total of 4.8 million reads (Supplementary Table 1) having an average length (across all flowcells) of 7 kb and totaling 33 Mb of sequence data (∼44× coverage given the estimated genome size), and sequencing each HiC library produced 150, 156, and 241 M read pairs (total: 547 M).

**Fig. 2.**
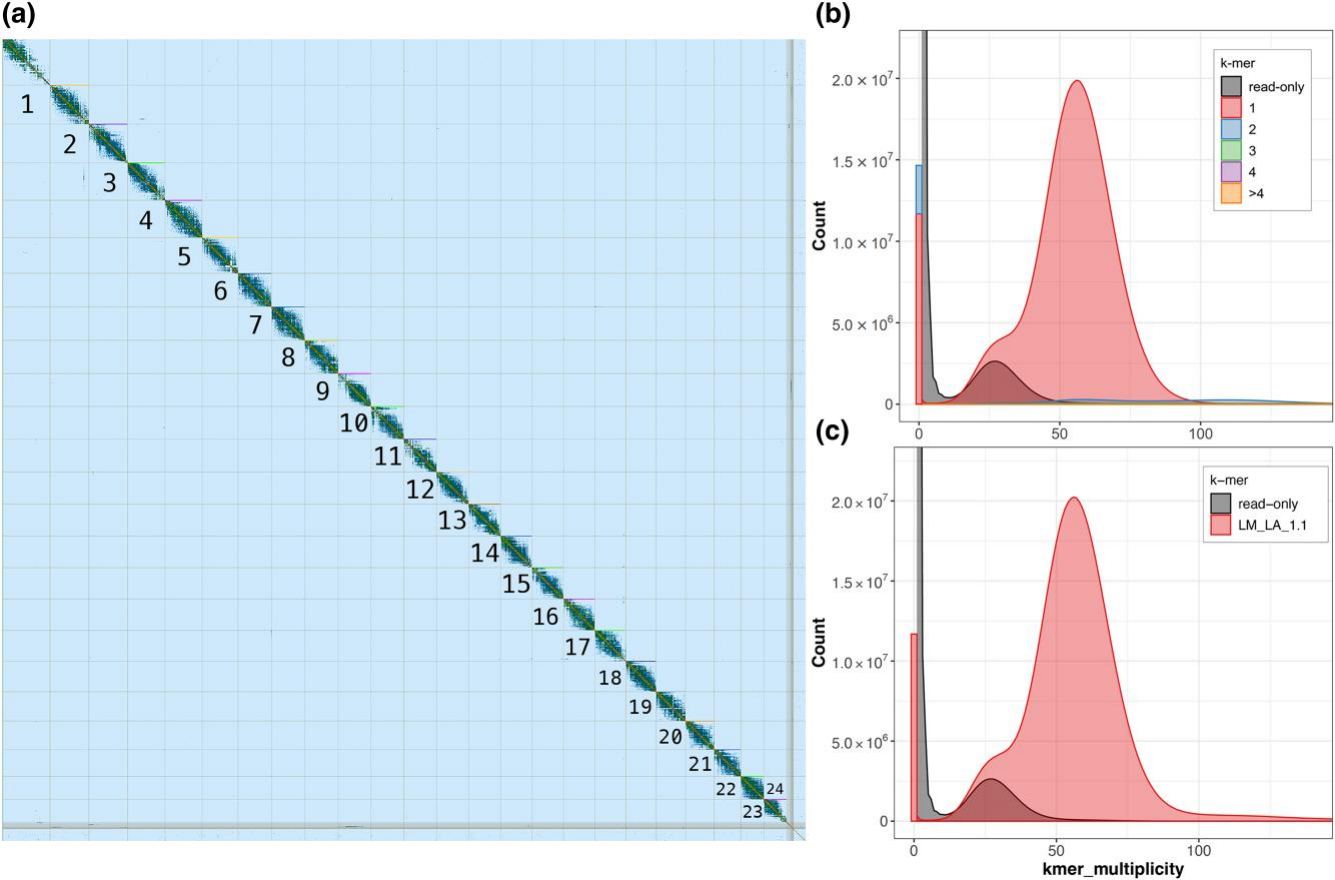
a) Contact map of the Lm_LA_1.1 assembly with the number of each scaffold placed below the corresponding portion of the map. The number for scaffold 24 is placed *above* the corresponding portion of the map to keep from obstructing the contacts of smaller contigs and scaffolds. Merqury copy number spectrum plot b) and assembly spectrum plot c).

The contig assembly produced by flye was more complete than that produced by wtdbg2 (Table 1), and short-read polishing of the flye contigs using Pilon corrected 1.3 million SNPs, 2.9 million small insertions, and 2.4 million small deletions totaling 7.4 million base pairs (∼1% of the total contig length). After trimming, 526 M HiC read pairs were aligned to the polished contigs using the juicer workflow, ∼382 M read pairs were unique, and the juicer software identified 290 M HiC contacts that were used to scaffold the assembly. After scaffolding and manually correcting the assembly using 3D-DNA and JuiceBox, contiguity and completeness metrics substantially improved (Table 1). Taxonomic partitioning using BlobTools did not identify any contigs that aligned to unexpected taxonomic groups, and the final rounds of filtering for short- and/or low-coverage contigs had minimal impact on contiguity and BUSCO metrics (Table 1) of the final assembly, which we refer to as Lm_LA_1.1. The final assembly contained 24 large scaffolds (scaffold1–scaffold24; >25 Mb; Fig. 2a), a number equal to the count of Bluegill chromosomes (Roberts 1964). The next largest scaffold (scaffold25) showed a substantial reduction in length (1.2 Mb), suggesting that it, and the remaining scaffolds, are unplaced components of the Bluegill chromosomes. Merqury estimated that assembly completeness was 92.5% and the consensus QV score was 31 (>99.9% accuracy). Copy number and assembly spectrum plots produced by Merqury are provided in Fig. 2, b and c.

**Table 1.** Contiguity statistics, assembly characteristics, and BUSCO scores for different stages of the *L. macrochirus* (Lm_LA_1.1).

	wtdbg2		flye		flye+3D-DNA		Final assembly Lm_LA_1.1 (+mtDNA)	
Sum	772,382,401	—	894,201,655	—	896,900,488	—	889,052,347	—
Count	2,220	—	6,105	—	3,556	—	1,841	—
Average length	347,920	—	146,470	—	252,222	—	482,918	—
Largest	16,197,694	—	13,778,814	—	52,096,719	—	52,096,719	—
N50 (L50)	3,537,726	(57)	1,327,969	(148)	36,130,255	(12)	36,129,979	(12)
N60 (L60)	2,263,457	(85)	903,957	(229)	35,377,480	(14)	35,377,416	(14)
N70 (L70)	1,597,059	(125)	548,450	(358)	34,736,361	(17)	34,736,361	(17)
N80 (L80)	854,351	(191)	292,041	(582)	33,525,000	(19)	33,525,000	(19)
N90 (L90)	305,142	(338)	114,766	(1,070)	29,519,818	(22)	29,519,818	(22)
N100 (L100)	1,830	(2,220)	230	(6,105)	77	(3,556)	1,000	(1,841)
N count	—	—	—	—	1,306,000	—	1,305,500	—
Gaps	—	—	—	—	2,612	—	2,611	—
eukaryota_odb10								
ȃComplete	204	80%	233	91%	253	99%	253	99%
ȃComplete single copy	204	80%	229	90%	245	96%	245	96%
ȃComplete duplicated	0	0%	4	2%	8	3%	8	3%
ȃFragmented	17	7%	17	7%	1	0%	1	0%
ȃMissing	34	13%	5	2%	1	0%	1	0%
ȃTotal	255	—	255	—	255	—	255	—
actinopterygii_odb10								
ȃComplete	—	—	—	—	—	—	3,578	98%
ȃComplete single copy	—	—	—	—	—	—	3,537	97%
ȃComplete duplicated	—	—	—	—	—	—	41	1%
ȃFragmented	—	—	—	—	—	—	20	1%
ȃMissing	—	—	—	—	—	—	42	1%
ȃTotal	—	—	—	—	—	—	3,640	

Repetitive elements identified using our de novo repeat models comprised 37% of the Lm_LA_1.1 assembly (Supplementary Table 2), which is a value similar to that estimated for other centrarchids (Sun *et al.* 2021). Retroelements and DNA transposons comprised approximately equal percentages (9%) of the total repeat content, and ∼15% of the total repeats were “unclassified.” Gene prediction using Braker2 with vertebrate protein sequences from OrthoDB identified a total of 76,741 possible gene regions, of which 17,233 were fully supported by external data.

The highly contiguous, chromosome-scale assembly we produced contributes to the growing number of genome assemblies representing the enormously diverse (Near *et al.* 2013) group of spiny-rayed fishes known as the acanthomorphs. Lm_LA_1.1 is the third assembly representing a centrarchid species that has been scaffolded to chromosome level (Supplementary Table 3) and the first assembly representing a member of the widespread sunfishes (*Lepomis* spp.). This assembly will facilitate studies of species relationships and species limits within this group, enable researchers to gain a better understanding of the degree and effects of introgression among *Lepomis* species, and serve as a tool to study the evolution of pelagic and benthic ecomorphs in a new organismal model.

## Supplementary Material

jkad019_Supplementary_Data

## Data Availability

All sequencing data and the final assembly, Lm_LA_1.1, are available from NCBI BioProject (PRJNA830889). Short-insert, Nanopore, and HiC reads are also available from the NCBI SRA (SRP372356), and the Whole Genome Shotgun project has been deposited at DDBJ/ENA/GenBank under the accession JALXJV000000000. The version described in this manuscript is version JALXJV020000000. The Supplemental Tables, a list of steps used to assemble and annotate the genome, PretextMap, Merqury results, RepeatMasker annotations, and gene predictions are available from FigShare (https://doi.org/10.6084/m9.figshare.21215777). Supplemental material available at G3 online.

## References

[jkad019-B1] Aday DD III , Parkos JJ, Wahl DH. Population and community ecology of Centrarchidae. In: Cook SJ, Philipp DP, editors. Centrarchid Fishes: Diversity, Biology, and Conservatio: 2009. p. 134–164. West Sussex, UK: Wiley-Blackwell.

[jkad019-B2] Arima Genomics . Arima Genomics Mapping Pipeline. 2019. [accessed 2022 Dec 15]. https://github.com/ArimaGenomics/mapping_pipeline/.

[jkad019-B3] Bolnick DI . Hybridization and speciation in centrarchids. In: Cook SJ, Philipp DP, editors. Centrarchid Fishes: Diversity, Biology, and Conservation; 2009. p. 39–69. West Sussex, UK: Wiley-Blackwell.

[jkad019-B4] Brůna T , Hoff KJ, Lomsadze A, Stanke M, Borodovsky M. 2021. BRAKER2: automatic eukaryotic genome annotation with GeneMark-EP+ and AUGUSTUS supported by a protein database. NAR Genom Bioinform. 3(1):lqaa108. doi:10.1093/nargab/lqaa108. PMC7787252 33575650

[jkad019-B5] Brůna T , Lomsadze A, Borodovsky M. GeneMark-EP+: eukaryotic gene prediction with self-training in the space of genes and proteins. NAR Genom Bioinform. 2020;2(2):lqaa026. doi:10.1093/nargab/lqaa026. PMC7222226 32440658

[jkad019-B6] Buchfink B , Xie C, Huson DH. Fast and sensitive protein alignment using DIAMOND. Nat Methods. 2015;12(1):59–60. doi:10.1038/nmeth.3176. 25402007

[jkad019-B7] Buckner JC , Sanders RC, Faircloth BC, Chakrabarty P. The critical importance of vouchers in genomics. Elife. 2021;10:e68264. doi:10.7554/eLife.68264. PMC8186901 34061026

[jkad019-B8] Burr BM . A new intergeneric hybrid combination in nature: *Pomoxis annularis* × *Centrarchus macropterus*. Copeia. 1974;1974(1):269–271. doi:10.2307/1443040.

[jkad019-B9] Dfam-Consortium. Dfam TE Tools Container . 2022. [accessed 2022 Oct 4]. https://github.com/Dfam-consortium/TETools.

[jkad019-B10] Dudchenko O , Batra SS, Omer AD, Nyquist SK, Hoeger M, Durand NC, Shamim MS, Machol I, Lander ES, Aiden AP, et al De novo assembly of the *Aedes aegypti* genome using Hi-C yields chromosome-length scaffolds. Science. 2017;356(6333):92–95. doi:10.1126/science.aal3327. 28336562 PMC5635820

[jkad019-B11] Durand NC , Robinson JT, Shamim MS, Machol I, Mesirov JP, Lander ES, Aiden EL. Juicebox provides a visualization system for Hi-C contact maps with unlimited zoom. Cell Syst. 2016;3(1):99–101. doi:10.1016/j.cels.2015.07.012. 27467250 PMC5596920

[jkad019-B12] Durand NC , Shamim MS, Machol I, Rao SS, Huntley MH, Lander ES, Aiden EL. Juicer provides a one-click system for analyzing loop-resolution Hi-C experiments. Cell Syst. 2016;3(1):95–98. doi:10.1016/j.cels.2016.07.002. 27467249 PMC5846465

[jkad019-B13] Faircloth BC . Braker2 Container. 2022. [accessed 2022 Sep 27]. https://github.com/faircloth-lab/singularity/tree/main/braker.

[jkad019-B14] Flynn JM , Hubley R, Goubert C, Rosen J, Clark AG, Feschotte C, Smit AF. Repeatmodeler2 for automated genomic discovery of transposable element families. Proc Natl Acad Sci U S A. 2020;117(17):9451–9457. doi:10.1073/pnas.1921046117. 32300014 PMC7196820

[jkad019-B15] Formenti G , Rhie A, Balacco J, Haase B, Mountcastle J, Fedrigo O, Brown S, Capodiferro MR, Al-Ajli FO, Ambrosini R, *et al*. Complete vertebrate mitogenomes reveal widespread repeats and gene duplications. Genome Biol. 2021;22. doi:10.1186/s13059-021-02336-9. PMC8082918 33910595

[jkad019-B16] Fricke R , Eschmeyer WN, Van der Laan R. Eschmeyer’s Catalog of Fishes: Genera, Species, References. 2022. [accessed 2022 Sep 27]. http://researcharchive.calacademy.org/research/ichthyology/catalog/fishcatmain.asp.

[jkad019-B17] Girard CF . Descriptions of new fishes, collected by Dr. A.L. Heermann, naturalist attached to the survey of the Pacific railroad route, under Lieut. R.S. Williamson, USA. Proc Acad Nat Sci Phila. 1854;7:129–165.

[jkad019-B18] Gotoh O . A space-efficient and accurate method for mapping and aligning cDNA sequences onto genomic sequence. Nucleic Acids Res. 2008;36(8):2630–2638. doi:10.1093/nar/gkn105. 18344523 PMC2377433

[jkad019-B19] Hoff KJ , Lange S, Lomsadze A, Borodovsky M, Stanke M. BRAKER1: unsupervised RNA-seq-based genome annotation with GeneMark-ET and AUGUSTUS. Bioinformatics. 2016;32(5):767–769. doi:10.1093/bioinformatics/btv661. 26559507 PMC6078167

[jkad019-B20] Hoff KJ , Lomsadze A, Borodovsky M, Stanke M. Whole-genome annotation with BRAKER. Methods Mol Biol. 2019;1962:65–95. doi:10.1007/978-1-4939-9173-0_5. 31020555 PMC6635606

[jkad019-B21] Iwata H , Gotoh O. Benchmarking spliced alignment programs including Spaln2, an extended version of Spaln that incorporates additional species-specific features. Nucleic Acids Res. 2012;40(20):e161. doi:10.1093/nar/gks708. 22848105 PMC3488211

[jkad019-B22] Jones P , Binns D, Chang HY, Fraser M, Li W, McAnulla C, McWilliam H, Maslen J, Mitchell A, Nuka G, et al Interproscan 5: genome-scale protein function classification. Bioinformatics. 2014;30(9):1236–1240. doi:10.1093/bioinformatics/btu031. 24451626 PMC3998142

[jkad019-B23] Kawamura K , Yonekura R, Katano O, Taniguchi Y, Saitoh K. Origin and dispersal of bluegill sunfish, *Lepomis macrochirus*, in Japan and Korea. Mol Ecol. 2006;15(3):612–621. doi:10.1111/j.1365-294X.2006.02823.x. 16499689

[jkad019-B24] Kent WJ , Sugnet CW, Furey TS, Roskin KM, Pringle TH, Zahler AM, Haussler D. The human genome browser at UCSC. Genome Res. 2002;12(6):996–1006. doi:10.1101/gr.229102. 12045153 PMC186604

[jkad019-B25] Kolmogorov M , Yuan J, Lin Y, Pevzner PA. Assembly of long, error-prone reads using repeat graphs. Nat Biotechnol. 2019;37(5):540–546. doi:10.1038/s41587-019-0072-8. 30936562

[jkad019-B26] Kriventseva EV , Kuznetsov D, Tegenfeldt F, Manni M, Dias R, Simão FA, Zdobnov EM. OrthoDB v10: sampling the diversity of animal, plant, fungal, protist, bacterial and viral genomes for evolutionary and functional annotations of orthologs. Nucleic Acids Res. 2019;47(D1):D807–D811. doi:10.1093/nar/gky1053. 30395283 PMC6323947

[jkad019-B27] Laetsch DR , Blaxter ML. BlobTools: interrogation of genome assemblies. F1000Research. 2017;6:1287. doi:10.12688/f1000research.12232.1.

[jkad019-B28] Li H. Aligning sequence reads, clone sequences and assembly contigs with BWA-MEM. 2013. doi:10.48550/arXiv.1303.3997

[jkad019-B29] Li H . Minimap2: pairwise alignment for nucleotide sequences. Bioinformatics. 2018;34:3094–3100. doi:10.1093/bioinformatics/bty191 29750242 PMC6137996

[jkad019-B30] Li H , Handsaker B, Wysoker A, Fennell T, Ruan J, Homer N, Marth G, Abecasis G, Durbin R, Proc GPD. The sequence alignment/map format and SAMtools. Bioinformatics. 2009;25(16):2078–2079. doi:10.1093/bioinformatics/btp352. 19505943 PMC2723002

[jkad019-B31] Lieberman-Aiden E , van Berkum NL, Williams L, Imakaev M, Ragoczy T, Telling A, Amit I, Lajoie BR, Sabo PJ, Dorschner MO, et al Comprehensive mapping of long-range interactions reveals folding principles of the human genome. Science. 2009;326(5950):289–293. doi:10.1126/science.1181369. 19815776 PMC2858594

[jkad019-B32] Lomsadze A , Ter-Hovhannisyan V, Chernoff YO, Borodovsky M. Gene identification in novel eukaryotic genomes by self-training algorithm. Nucleic Acids Res. 2005;33(20):6494–6506. doi:10.1093/nar/gki937. 16314312 PMC1298918

[jkad019-B33] Maezono Y , Miyashita T. Community-level impacts induced by introduced largemouth bass and bluegill in farm ponds in Japan. Biol Conserv. 2003;109(1):111–121. doi:10.1016/S0006-3207(02)00144-1.

[jkad019-B34] Manni M , Berkeley MR, Seppey M, Simão FA, Zdobnov EM. BUSCO update: novel and streamlined workflows along with broader and deeper phylogenetic coverage for scoring of eukaryotic, prokaryotic, and viral genomes. Mol Biol Evol. 2021;38(10):4647–4654. doi:10.1093/molbev/msab199. 34320186 PMC8476166

[jkad019-B35] Marcais G , Kingsford C. A fast, lock-free approach for efficient parallel counting of occurrences of k-mers. Bioinformatics. 2011;27(6):764–770. doi:10.1093/bioinformatics/btr011. 21217122 PMC3051319

[jkad019-B36] Nakabayashi R , Morishita S. HiC-Hiker: a probabilistic model to determine contig orientation in chromosome-length scaffolds with Hi-C. Bioinformatics. 2020;36(13):3966–3974. doi:10.1093/bioinformatics/btaa288. 32369554 PMC7672694

[jkad019-B37] Ndaleni PM , Wasserman RJ, Ellender BR, Wyl OLF. Diet of bluegill *Lepomis macrochirus* in a South African reservoir during winter and summer. Afr J Aquat Sci. 2018;43(1):85–88. doi:10.2989/16085914.2018.1436514.

[jkad019-B38] Near TJ , Bolnick DI, Wainwright PC. Investigating phylogenetic relationships of sunfishes and black basses (Actinopterygii: Centrarchidae) using DNA sequences from mitochondrial and nuclear genes. Mol Phylogenet Evol 2004;32(1):244–257. doi:10.1016/j.ympev.2003.12.010. 15186819

[jkad019-B39] Near TJ , Bolnick DI, Wainwright PC. Fossil calibrations and molecular divergence time estimates in centrarchid fishes (Teleostei: Centrarchidae). Evolution. 2005;59(8):1768–1782. doi:10.1111/j.0014-3820.2005.tb01825.x. 16329246

[jkad019-B40] Near TJ , Dornburg A, Eytan RI, Keck BP, Smith WL, Kuhn KL, Moore JA, Price SA, Burbrink FT, Friedman M, et al Phylogeny and tempo of diversification in the superradiation of spiny-rayed fishes. Proc Natl Acad Sci U S A. 2013;110(31):12738–12743. doi:10.1073/pnas.1304661110. 23858462 PMC3732986

[jkad019-B41] Near TJ , Kim D. Phylogeny and time scale of diversification in the fossil-rich sunfishes and black basses (Teleostei: Percomorpha: Centrarchidae). Mol Phylogenet Evol. 2021;161:107156. doi:10.1016/j.ympev.2021.107156. 33741536

[jkad019-B42] Near TJ , Koppelman JB. Species diversity, phylogeny and phylogeography of Centrarchidae. In: Cook SJ, Philipp DP, editors. Centrarchid Fishes: Diversity, Biology, and Conservation; 2009. p. 1–38. West Sussex, UK: Wiley-Blackwell.

[jkad019-B43] Page LM , Burr BM. Peterson field guide to freshwater fishes of North America north of Mexico. Boston/New York: Houghton Mifflin Harcourt. 2011.

[jkad019-B44] QIAGEN . 2015. QIAGEN Genomic DNA Handbook. Venlo, The Netherlands: Qiagen NV. p. 70.

[jkad019-B45] Quinlan AR , Hall IM. BEDTools: a flexible suite of utilities for comparing genomic features. Bioinformatics. 2010;26(6):841–842. doi:10.1093/bioinformatics/btq033. 20110278 PMC2832824

[jkad019-B46] Rafinesque CS . Prodrome de 70 nouveaux genres d’animaux découverts dans l’intérieur des États-Unis d’Amérique, durant l’année 1818. J Phys Chim Hist Natl Arts. 1819;88:417–429.

[jkad019-B47] Ragland CJ , Gold JR. Genome size variation in the North American sunfish genus Lepomis (Pisces: Centrarchidae). Genet Res (Camb). 1989;53(3):173–182. doi:10.1017/S0016672300028135.

[jkad019-B48] Regier HA . On the evolution of bass-bluegill stocking policies and management recommendations. Prog Fish-Cult. 1962;24(3):99–111. doi:10.1577/1548-8659(1962)24[99:OTEOBS]2.0.CO;2.

[jkad019-B49] Rhie A , Walenz BP, Koren S, Phillippy AM. Merqury: reference-free quality, completeness, and phasing assessment for genome assemblies. Genome Biol. 2020;21(1):245. doi:10.1186/s13059-020-02134-9. 32928274 PMC7488777

[jkad019-B50] Roberts FL . A chromosome study of twenty species of Centrarchidae. J Morphol. 1964;115(3):401–417. doi:10.1002/jmor.1051150305. 14234867

[jkad019-B51] Rohland N , Reich D. Cost-effective, high-throughput DNA sequencing libraries for multiplexed target capture. Genome Res. 2012;22(5):939–946. doi:10.1101/gr.128124.111. 22267522 PMC3337438

[jkad019-B52] Ruan J , Li H. Fast and accurate long-read assembly with wtdbg2. Nat Methods. 2020;17(2):155–158. doi:10.1038/s41592-019-0669-3. 31819265 PMC7004874

[jkad019-B53] Rundle HD , Nagel L, Boughman JW, Schluter D. Natural selection and parallel speciation in sympatric sticklebacks. Science. 2000;287(5451):306–308. doi:10.1126/science.287.5451.306. 10634785

[jkad019-B54] Salter JF , Johnson O, Stafford NJ, Herrin WF, Schilling D, Cedotal C, Brumfield RT, Faircloth BC. A highly contiguous reference genome for Northern Bobwhite (*Colinus virginianus*). G3 (Bethesda). 2019;9(12):3929–3932. doi:10.1534/g3.119.400609. 31611345 PMC6893191

[jkad019-B55] Shen W , Le S, Li Y, Hu F. SeqKit: A cross-platform and ultrafast toolkit for FASTA/Q file manipulation. PLoS ONE. 2016;11. doi: 10.1371/journal.pone.0163962 PMC5051824 27706213

[jkad019-B56] Smith AFA , Hubley R, Green P. RepeatMasker Open-4.0. 2022. [accessed 2022 Oct 4]. https://www.repeatmasker.org.

[jkad019-B57] Stanke M , Diekhans M, Baertsch R, Haussler D. Using native and syntenically mapped cDNA alignments to improve de novo gene finding. Bioinformatics. 2008;24(5):637–644. doi:10.1093/bioinformatics/btn013. 18218656

[jkad019-B58] Stanke M , Schöffmann O, Morgenstern B, Waack S. Gene prediction in eukaryotes with a generalized hidden Markov model that uses hints from external sources. BMC Bioinform. 2006;7(1):62. doi:10.1186/1471-2105-7-62. PMC1409804 16469098

[jkad019-B59] Sun C , Li J, Dong J, Niu Y, Hu J, Lian J, Li W, Li J, Tian Y, Shi Q, et al Chromosome-level genome assembly for the largemouth bass *Micropterus salmoides* provides insights into adaptation to fresh and brackish water. Mol Ecol Resour. 2021;21(1):301–315. doi:10.1111/1755-0998.13256. 32985096

[jkad019-B60] Uchii K , Okuda N, Yonekura R, Karube Z, Matsui K, Kawabata Z. Trophic polymorphism in bluegill sunfish (*Lepomis macrochirus*) introduced into Lake Biwa: evidence from stable isotope analysis. Limnology. 2007;8(1):59–63. doi:10.1007/s10201-006-0196-7.

[jkad019-B61] Vurture GW , Sedlazeck FJ, Nattestad M, Underwood CJ, Fang H, Gurtowski J, Schatz MC. Genomescope: fast reference-free genome profiling from short reads. Bioinformatics. 2017;33(14):2202–2204. doi:10.1093/bioinformatics/btx153. 28369201 PMC5870704

[jkad019-B62] Walker BJ , Abeel T, Shea T, Priest M, Abouelliel A, Sakthikumar S, Cuomo CA, Zeng Q, Wortman J, Young SK, et al Pilon: an integrated tool for comprehensive microbial variant detection and genome assembly improvement. PLoS One. 2014;9(11):e112963. doi:10.1371/journal.pone.0112963. 25409509 PMC4237348

[jkad019-B63] Wellcome Sanger Institute . assembly-stats. 2022a. [accessed 2022 Oct 4]. https://github.com/sanger-pathogens/assembly-stats.

[jkad019-B64] Wellcome Sanger Institute . PretextMap https://github.com/wtsi-hpag/PretextMap and PretextView. 2022b. [accessed 2022 Dec 15]. https://github.com/wtsi-hpag/PretextView.

[jkad019-B65] Yamamoto MN . 1992. Occurrence, distribution and abundance of accidentally introduced freshwater aquatic organisms in Hawaii. State of Hawaii, Federal Aid in Sportfish Restoration, Dingell-Johnson JOR. Freshwater Fisheries Research and Surveys, Project No. F-14-R-16. US Federal Government Document.

